# The cutaneous sympathetic blockade associated with labour epidural analgesia: a quasi-experimental study conducted during labour and after delivery^☆^

**DOI:** 10.1016/j.bja.2025.07.077

**Published:** 2025-09-09

**Authors:** Giulia M.V. Iacona, Aimee R. Rolph, Hugo F.M. Manteigas, Paul H. Strutton, David A. Low, Christopher J. Mullington

**Affiliations:** 1 MSk Lab, Imperial College London, London, UK; 2 Theatres and Anaesthetics, Imperial College Healthcare NHS Trust, London, UK; 3 Sport and Exercise Sciences, Liverpool John Moores University, Liverpool, UK

**Keywords:** body temperature, epidural analgesia, epidural-related maternal fever, epidural-related maternal hyperthermia, labour, pregnancy, sympathetic nervous system, thermoregulation

## Abstract

**Background:**

The mechanisms contributing to epidural-related maternal hyperthermia remain unclear. One explanation is that blockade of cholinergic sympathetic nerves prevents active vasodilation and sweating. However, it is not known how labour epidural analgesia affects cutaneous sympathetic function. The aim of this study was to test the hypothesis that labour epidural analgesia inhibits cholinergic and noradrenergic function in the lower, but not the upper, limbs.

**Methods:**

Twenty women (mean age [range]:33 yr [21-48]) receiving epidural analgesia had upper and lower limb cutaneous sympathetic skin responses assessed during labour (epidural) and after delivery (control). Responses were evoked with auditory stimuli delivered through headphones. Sudomotor skin responses (cholinergic function) were recorded with Ag/AgCl electrodes on the hand and foot (median [range]). Vasomotor skin responses (noradrenergic function) were recorded with laser Doppler flowmetry on the finger and toe (median [range]).

**Results:**

Sudomotor skin response amplitude was less during labour in both the hand (epidural: 0.05 mV [0.00–1.87] *vs* control: 0.69 mV [0.02–3.73]; *P*=0.013) and the foot (epidural: 0.00 mV [0.00–0.92] *vs* control: 0.53 mV [0.05–2.79]; *P*<0.001). Vasomotor skin response reduction rate was less during labour in the toe (epidural: 6.3% [0.0–41.8] *vs* control: 18.2% [0.0–53.3]; *P*<0.001) but was not different between visits in the finger (epidural: 7.9% [0.0-29.9] *vs* control: 5.0% [0.0-29.8]; *P*=0.242).

**Conclusions:**

Labour epidural analgesia can inhibit cholinergic sympathetic outflow to 90% of the body surface. Cholinergic sympathetic blockade could prevent women offloading heat generated during labour. The distribution of cutaneous sympathetic blockade varied among individuals. Cholinergic sympathetic blockade distribution is a potential contributing factor to epidural-related maternal hyperthermia.

**Editor’s key points**

• The mechanisms contributing to epidural-related maternal hyperthermia remain unclear.
• Maternal hyperthermia could result from blockade of cholinergic sympathetic nerves that prevents active vasodilation and sweating; however, this hypothesis has never been tested.
• The authors quantified upper and lower limb cutaneous sympathetic skin responses during labour and again after delivery.
• Epidural analgesia during labour inhibited cholinergic sympathetic outflow to 90% of the body surface.
• Cholinergic sympathetic blockade distribution is a potential contributing factor to epidural-related maternal hyperthermia.

Labour epidural analgesia increases the risk of intrapartum hyperthermia (core temperature >38°C).^1^ Intrapartum hyperthermia is associated with adverse maternal and neonatal outcomes, including antibiotic usage, operative delivery, neonatal ICU admission, and neonatal brain injury.^1^, ^2^, ^3^, ^4^ The mechanism underlying epidural-related maternal hyperthermia (ERMH) is unclear, but a leading theory is cutaneous sympathetic blockade.^5^^,^^6^ The cutaneous sympathetic supply regulates body temperature through active vasoconstriction, active vasodilation, and sweating.^7^^,^^8^ Therefore, blockade of the sympathetic supply to a proportion of the body surface could impair women’s ability to lose the additional heat generated during labour, resulting in an increase in body temperature.^5^^,^^6^

Humans have a dual cutaneous sympathetic supply. The noradrenergic pathway is responsible for active vasoconstriction, and is active during cold stress and in thermoneutral conditions.^7^ The cholinergic pathway is responsible for active vasodilation and sweating, and is active during heat stress.^7^^,^^8^ During labour, the metabolism of the fetus and the contraction of uterine and skeletal muscle increase heat production.^6^^,^^9^^,^^10^ Therefore, labour is a form of heat stress and, when environmental temperature is elevated to the recommended 25–28°C, it is likely that cholinergic sympathetic activity is required to maintain heat balance and prevent hyperthermia.^11^^,^^12^

The impact of neuraxial blockade on body temperature is dependent upon the thermal state of the recipient before block initiation. Before elective Caesarean section and non-obstetric surgery, patients are in a thermoneutral state. Consequently, neuraxial blockade inhibits active vasoconstriction, increasing skin blood flow and cutaneous heat loss, and causing body temperature to decrease.^13^ In contrast, upper limb regional anaesthesia during established hyperthermia decreases skin blood flow,^7^^,^^14^ and after epidural extension for intrapartum Caesarean section (a heat stress scenario) cutaneous heat loss decreases and body temperature increases.^6^ It is not known how labour epidural analgesia affects cutaneous sympathetic function.

This single-centre physiological study aimed to assess the impact of labour epidural analgesia on cutaneous sympathetic function in the upper and lower limbs. To provide analgesia for uterine contractions, labour epidural analgesia must, at a minimum, block pain sensation in the T10–L2 distribution.^15^ The cutaneous sympathetic supply to the upper and lower limbs exits the vertebral column between T2–T8 and T10–L2, respectively.^15^ Therefore, it was hypothesised that labour epidural analgesia would inhibit both cholinergic and noradrenergic cutaneous sympathetic function in the lower limbs but not the upper limbs.

## Methods

### Study design

Ethical approval was obtained from London Fulham Research Ethics Committee (13/LO/0672).

A convenience sample of potential participants was approached by the study team after epidural catheter insertion. After reading the participant information sheet, participants were screened for eligibility and given the opportunity to ask questions. Participants who fulfilled the inclusion and exclusion criteria and were willing to participate provided oral and written consent.

### Inclusion and exclusion criteria

Women aged ≥18 yr with effective labour epidural analgesia were included. Women were excluded if they had any cardiovascular or neurological disease, were taking adrenoreceptor blocking medications, or were unable to speak English.

### Study procedure

**Fig 1:**
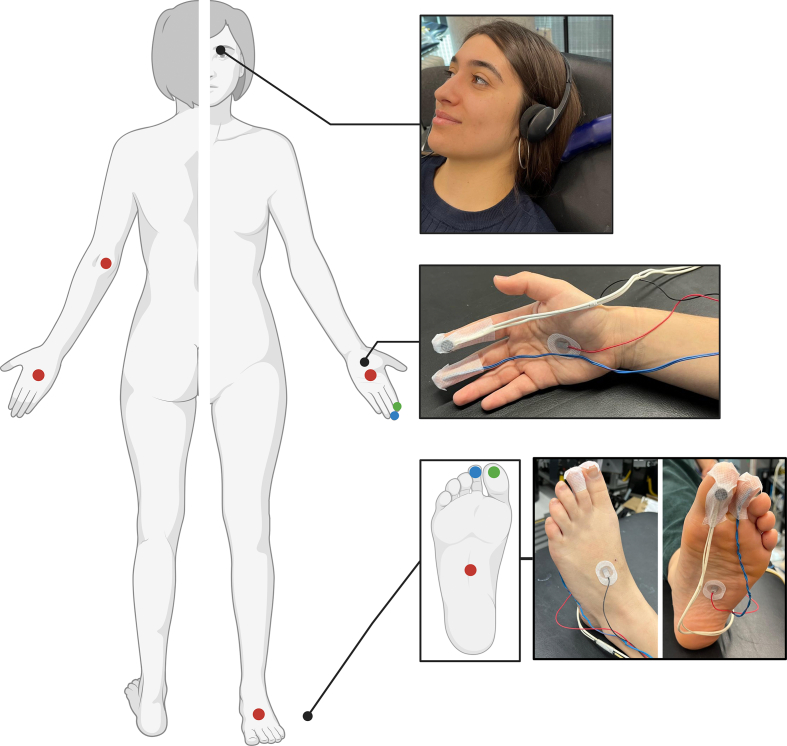
Schematic of the study apparatus. Red dots = surface electrodes (sudomotor skin responses). Green dots = laser Doppler flowmetry probes (vasomotor skin responses). Blue dots = thermocouples (skin temperature). (Created in BioRender. Iacona, G. [2025] https://BioRender.com/t60b904). Subject in picture is part of the research team who gave full permission for photograph to be used.

Participants undertook the experimental protocol on two occasions: during labour (epidural visit) and after delivery (control visit). During the epidural visit, participants were in the first stage or the passive component of the second stage of labour (regular, painful contractions resulting in cervical dilation) and had effective epidural analgesia *in situ* for >3 h (to ensure block distribution stability). Epidural analgesia was administered with midwife-delivered boluses of levobupivacaine 0.1% plus fentanyl 2 μg ml^−1^. The control visit was conducted within 48 h of delivery, after epidural catheter removal and neuraxial blockade resolution (walking unaided and urination after urinary catheter removal). At each visit, a standardised assessment procedure was followed. First, the distribution of sensory and motor blockade was assessed. Then, the study apparatus was attached (Fig. 1) and cutaneous sympathetic function assessed. Ambient noise was minimised during testing.

### Measurements

Noradrenergic and cholinergic sympathetic function was assessed with vasomotor and sudomotor evoked skin responses, respectively. Evoked skin responses are an established method of quantifying cutaneous sympathetic function in health and disease.^16^^,^^17^ An evoked skin response consists of a neuronal stimulus which activates both the noradrenergic and cholinergic efferent sympathetic pathways simultaneously via a brainstem reflex.^16^ Noradrenergic pathway activation results in vasoconstriction which is recorded on the glabrous skin of the hand and foot.^17^ Cholinergic pathway activation results in sweating which is detected through electrodermal potential changes.^16^ In the present study, skin responses were evoked with auditory stimuli, as this is the least invasive of the potential stimulus modalities.^16^^,^^18^ During each assessment, participants received 10 auditory stimuli (0.1 ms, 120 Hz, 100–120 dB) delivered through headphones (RP-HT225; Panasonic, Kadoma, Japan).^18^ Data were recorded for 25 s pre-stimulus and 35 s post-stimulus. The inter-stimulus interval was >60 s to prevent habituation.^19^ If external noise was detected in the peristimulus period, the stimulus was repeated and the contaminated stimulus excluded. Vasomotor skin responses were recorded with laser Doppler flowmetry probes (457 Small Angled Thermostatic Probe; Perimed Instruments, Järfälla, Sweden) attached to the palmar surface of the index finger and to the plantar surface of the great toe.^17^ Sudomotor responses were recorded with pairs of self-adhesive electrodes (Ag/AgCl electrodes; Neuroline 715, Ambu, Ballerup, DK) attached to the hand (palm and dorsum) and the foot (plantar and dorsum). A reference electrode was attached to the skin overlying the olecranon. Electrodermal potentials were filtered (0.1–100 Hz) and amplified (x100; D-360 Isolated Patient Amplifier; Digitimer, Welwyn Garden City, UK).^19^ All apparatus was connected to the opposite side of the body to the participant’s i.v. cannula to mitigate i.v. fluid temperature artifact. Because thermal state influences sympathetic skin responses, sublingual core temperature (maximum of three recordings; SureTemp Plus; Welch Allyn, Skaneateles Falls, NY, USA) and finger and toe skin temperature (T-type thermocouple/thermocouple meter; TC-2000; Sable Systems, Las Vegas, NV, USA) were recorded during sympathetic function assessments.^16^^,^^17^ Sympathetic skin responses and skin temperatures were sampled at 2 kHz using a data acquisition system (1401+; Cambridge Electronic Design [CED], Cambridge, UK) and Signal software (version 7.06; CED). Data were processed offline using bespoke scripts. Room temperature and humidity were recorded with a data logger (RS-172; RS Components, Corby, UK). Cold sensation was assessed with ethyl chloride (the point at which the spray first felt icy), testing from the lower abdomen in the cephalad and caudad directions.^20^ Lower limb motor function was assessed with a 4-point score (4, full movement of legs and feet; 3, no hip flexion but movement of the knee and feet; 2, no knee flexion but movement of feet; 1, no movement of legs or feet).^21^ Clinical data were extracted from the electronic patient record, including patient characteristics, obstetric history, labour characteristics, maximum intrapartum core temperature, maternal outcomes, and neonatal outcomes. In the study centre, core temperature is recorded every 4 h during labour, and every 1 h if maternal sepsis is suspected.

### Primary outcome

The *a priori*-defined primary outcome was the sudomotor skin response amplitude.

### Secondary outcomes

**Fig 2:**
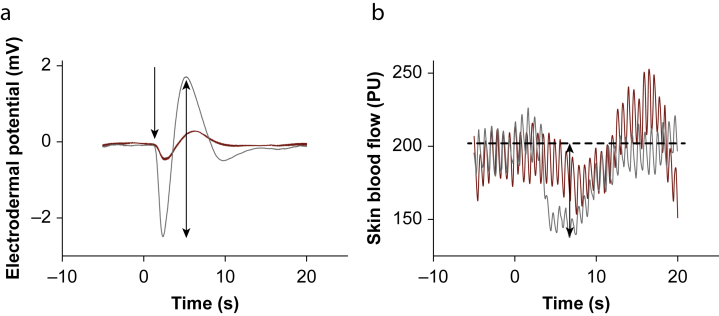
Example (a) sudomotor, and (b) vasomotor skin responses. Red traces are the epidural visit and grey traces are the control visit. Auditory stimuli were delivered at 0 s. The unidirectional arrow indicates the sudomotor skin response onset. The dashed line indicates the average pre-stimulus skin blood flow. Bidirectional arrows indicate the sudomotor skin response peak-to-peak amplitude and the vasomotor skin response reduction rate.

Secondary outcomes were vasomotor skin response reduction rates, sudomotor skin response latencies, number of participants with absent sudomotor and vasomotor skin responses, and sensory and motor blockade distribution on the ipsilateral side to the sympathetic skin responses. Sudomotor skin response amplitude was defined as the peak-to-peak amplitude (Fig. 2a).^16^ Vasomotor skin response reduction rate was defined as the reduction of skin blood flow seen after each stimulus as a percentage of the pre-stimulus value (Fig. 2b).^22^ Sudomotor skin response latency was defined as the interval between the stimulus and the first positive or negative deflection. Sudomotor skin response amplitude and vasomotor skin response reduction rate were calculated as the mean of all 10 stimuli.^16^^,^^22^ When a response was absent, the amplitude or reduction rate was considered to be 0. Sudomotor skin response latency was calculated as the mean of the stimuli when a response was evoked.^16^

### Statistical analysis

Given the lack of an *a priori* biologically relevant standard for the primary outcome, the sample size of 20 was a pragmatic choice based on similar studies in which eight to 20 participants were studied.^13^ To examine the adequacy of the study sample, a *post hoc* power calculation was performed with G∗Power software (version 3.1.9.6; Heinrich-Heine-Universität Düsseldorf, Kiel, Germany), which revealed a power of 0.89 for the between-visit main effect comparison of sudomotor skin response amplitude (Supplementary material). Data are presented as number (%), mean (range or sd), or median (range). Statistical analysis was performed with GraphPad Prism software (version 10.4.1; Dotmatics, Boston, MA, USA). Normality of data and equality of variance were examined with Shapiro–Wilk and Brown–Forsythe tests, respectively. Between-visits comparisons were conducted with two-way repeated measures analysis of variance (anova; sudomotor skin response amplitude), Wilcoxon tests (vasomotor skin response reduction rate), paired *t*-tests (core and skin temperatures, room temperature, and humidity), two-way mixed-effects anova (sudomotor skin response latency), and Fisher’s exact test (number of participants with absent sudomotor and vasomotor skin responses). The relationships between body temperatures (core, finger, toe) and sympathetic skin responses (sudomotor response amplitude, vasomotor response reduction rate) were analysed with Spearman rank correlation of the between-visit change in each variable.^17^ *Post hoc* analysis was conducted with Holm–Sidak methodology. *Post hoc P*-values are multiple comparison adjusted; *P*<0.05 was considered statistically significant.

## Results

### Study participants

Twenty women (mean age [range]: 33 yr [21-48]) receiving epidural catheters during labour were recruited from 23^rd^ March to 25^th^ May 2022 (Table 1). Epidural analgesia resulted in sensory and motor blockade, as determined by ethyl chloride and a 4-point score respectively (Table 2).^20^, ^21^ Sudomotor (hand) and vasomotor (finger) skin responses during labour and after delivery are displayed in Figure 2.

**Table 1:** Maternal and neonatal clinical data. ∗Indication = intrapartum sepsis.

Maternal characteristics	
Age (yr), mean (range)	33 (21–48)
Booking BMI (kg m^−2^), median (range)	23 (18–42)
Race, *n* (%)	
- White	8 (40)
- Black	6 (30)
- Asian	1 (5)
- Mixed	2 (10)
- Other	3 (15)
Parity, median (range)	1 (0–1)
Gestation <37 weeks, *n* (%)	1 (5)
Twins, *n* (%)	1 (5)
Comorbidities, *n* (%)	
- Gestational diabetes mellitus	2 (10)
- Pre-eclampsia	1 (5)
- Hepatitis B	1 (5)
**Labour descriptors**	
Labour onset (spontaneous), *n* (%)	9 (45)
Labour augmentation, *n* (%)	16 (80)
Labour duration (min), median (range)	621 (207–1487)
Total levobupivacaine 0.1% plus fentanyl 2 μg ml^−1^ (ml), median (range)	95 (50–215)
**Maternal clinical outcomes, *n* (%)**	
Sepsis evaluation	4 (20)
Antibiotics∗	4 (20)
Mode of delivery	
- Spontaneous	6 (30)
- Instrumental	3 (15)
- Caesarean	11 (55)
**Neonatal clinical outcomes, *n* (%)**	
Apgar score <7	
- 1 min	2 (10)
- 5 min	1 (5)
Umbilical artery pH <7.2	4 (19)
Sepsis evaluation	4 (19)
Antibiotics	4 (19)
Neonatal ICU admission	3 (14)

**Table 2:** Sudomotor and vasomotor skin responses, the distribution of sensory/motor blockade, and body temperatures during labour and after delivery. ∗On the ipsilateral side to the sympathetic skin responses.

	Epidural visit	Control visit	*P*-value
Visit timing (min), median (range)			–
- Epidural initiation: visit interval	332 (204–631)	–	
- Delivery: visit interval	–	1209 (807–1938)	
Cervical dilation (cm), median (range)	3 (1–10)	–	–
Environment, mean (sd)			
- Room temperature (°C)	24.9 (1.7)	25.4 (1.6)	0.251
- Humidity (%)	44 (7)	40 (10)	0.051
**Sudomotor skin responses**			
Peak-to-peak amplitude (mV), median (range)			
- Hand	0.05 (0.00–1.87)	0.69 (0.02–3.73)	0.016
- Foot	0.00 (0.00–0.92)	0.53 (0.05–2.79)	0.026
Latency (s), mean (sd)			0.713
- Hand	1.58 (0.20)	1.66 (0.21)	
- Foot	2.17 (0.27)	2.15 (0.32)	
No response, n (%)			
- Hand	8 (40%)	0 (0%)	0.003
- Foot	12 (60%)	0 (0%)	<0.001
**Vasomotor skin responses**			
Reduction rate (%), median (range)			
- Finger	7.9 (0.0–29.9)	5.0 (0.0–29.8)	0.242
- Toe	6.3 (0.0–41.8)	18.2 (0.0–53.3)	<0.001
No response, n (%)			
- Finger	2 (10%)	5 (25%)	0.408
- Toe	7 (35%)	2 (10%)	0.127
**Distribution of sensory and motor blockade, median (range)**			
Cold sensory level∗			
- Upper (thoracic)	8 (4–10)	–	–
- Lower (sacral)	5 (1–5)	–	–
Lower limb motor∗	4 (3–4)	4 (4–4)	–
**Body temperatures, mean (sd)**			
Core temperature (°C)	37.2 (0.3)	36.7 (0.4)	0.003
Skin temperature (°C)			
- Finger	33.7 (3.0)	34.9 (2.0)	0.216
- Toe	32.2 (3.1)	32.6 (2.6)	0.558

### Primary outcome: sudomotor skin response amplitude

**Fig 3:**
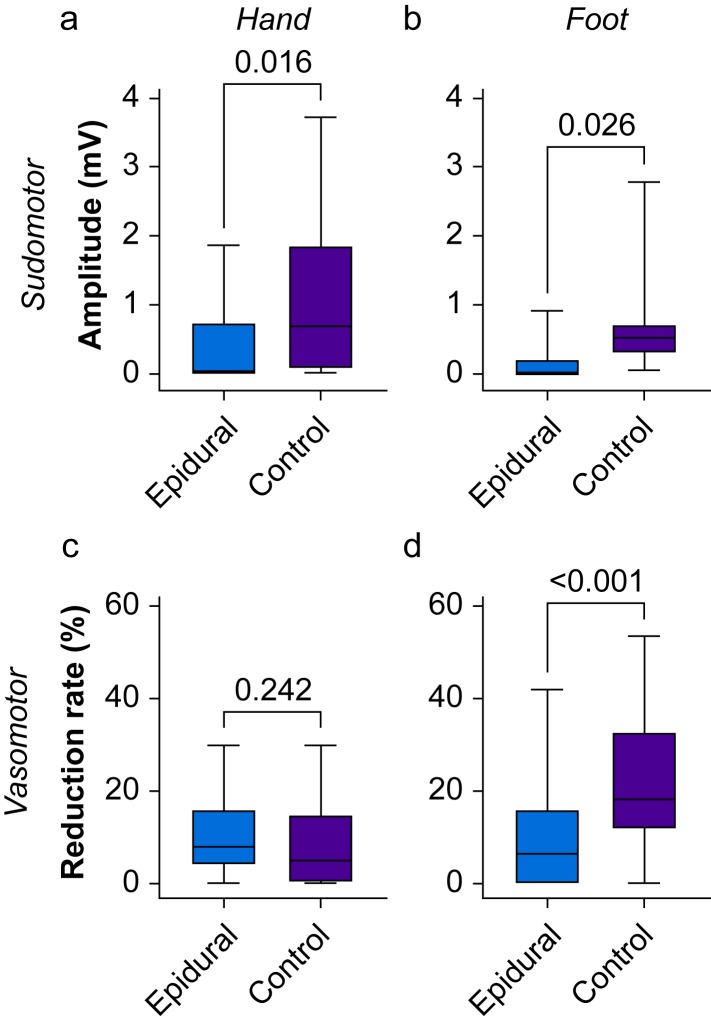
Box plots of sudomotor skin response amplitudes (a, hand; b, foot) and vasomotor skin response reduction rates (c, index finger; d, great toe) during labour (epidural visit) and after delivery (control visit). Boxes denote interquartile range and the line within each box denotes the median. Whiskers denote range. *P*<0.05 was considered statistically significant.

Sudomotor skin response amplitude was lower during labour in both the hand and the foot, compared with after delivery (Table 2 and Fig. 3).

### Secondary outcomes

Hand and foot sudomotor skin response latency did not differ between visits. The number of participants with absent sudomotor skin responses was greater on the epidural visit in both the hand and the foot. Vasomotor skin response reduction rate did not differ between visits in the finger but was less during labour in the toe (Table 2 and Fig. 3). The number of participants with absent vasomotor skin responses did not differ between visits in either the finger or the toe. The relationship between sudomotor and vasomotor blockade is summarised in Supplementary Table 1.

**Fig 4:**
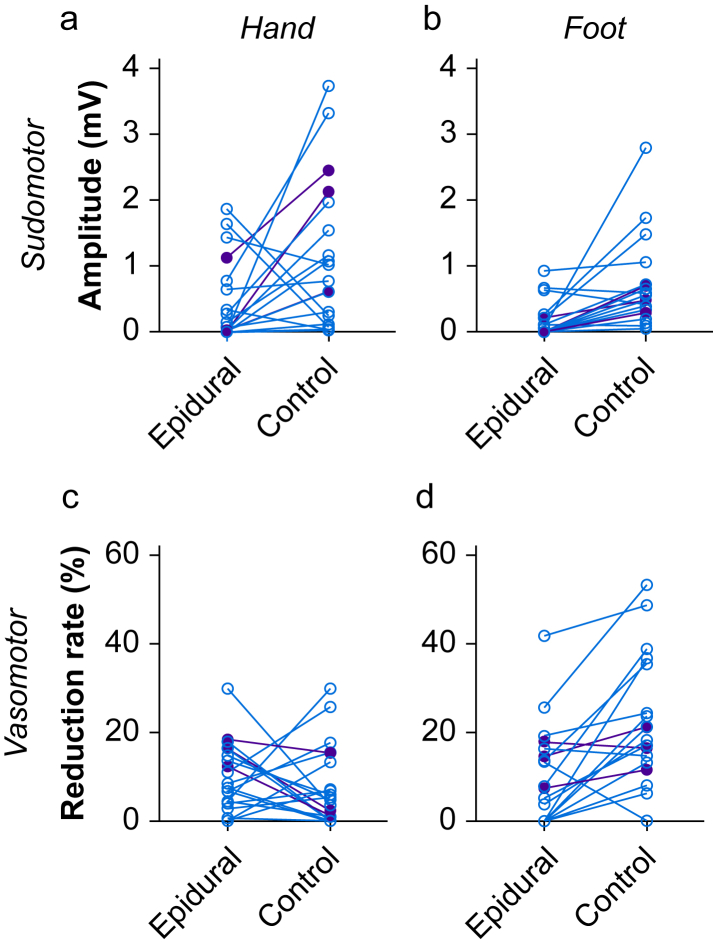
Individual participant sudomotor skin response amplitudes (a, hand; b, foot) and vasomotor skin response reduction rates (c, index finger; d, great toe) during labour (epidural visit) and after delivery (control visit). Purple symbols indicate women who had a maximum intrapartum core temperature >38°C. Fig 3, Fig 4 present the same data in different formats.

Core temperature was higher on the epidural visit. Finger and toe temperatures did not differ between visits. Three (15%) women had a maximum intrapartum core temperature >38°C. All three of these women had reduced sudomotor skin responses in the hand and foot on the epidural visit (Fig. 4). The variation in body temperature between visits was not correlated with the between-visit change in either sudomotor skin response amplitude or vasomotor skin response reduction rate (Supplementary Table 2).

## Discussion

This study investigated the distribution of cutaneous sympathetic blockade associated with labour epidural analgesia using evoked skin responses. As predicted, both cholinergic and noradrenergic sympathetic function were inhibited in the lower limbs. The inhibition of upper limb cholinergic sympathetic function was not anticipated.

To our knowledge, this is the first study to use evoked skin responses to quantify the cutaneous sympathetic blockade associated with any form of obstetric neuraxial blockade. The sudomotor skin response results align with previous investigations in non-obstetric scenarios.^23^, ^24^, ^25^, ^26^ Lumbar epidural blockade in healthy participants either eliminates or significantly reduces foot sudomotor skin response amplitude in 40–100% of participants,^23^, ^24^, ^25^ and when the upper sensory level is above T6, hand sudomotor skin response amplitude is either eliminated or reduced in 84% of participants.^25^ A similar pattern is observed during spinal anaesthesia for transurethral surgery (T2–T7 upper sensory level) where foot sudomotor skin responses are almost completely eliminated and hand responses are either eliminated or significantly reduced in 35% of patients.^26^ Vasomotor skin responses have not been used previously to assess noradrenergic sympathetic function during neuraxial blockade. However, the presence of finger vasoconstriction in response to cold and pain stimuli has been used to determine the upper level of sensory blockade during spinal anaesthesia.^27^

The inhibition of sudomotor function in the hand was not predicted. The cutaneous sympathetic fibres that innervate the upper limb exit the vertebral column between T2 and T8, and the sensory fibres from the uterus enter the vertebral column between T10 and L2.^15^ When labour epidural analgesia is administered via intermittent boluses, T10 is the minimum upper sensory level for effective analgesia.^28^ A prerequisite for the epidural visit was that participants were pain-free, but the bolus-visit interval was not standardised. Consequently, the upper cold sensation blockade level varied between T4 and T10. Because of the differing characteristics of cold sensory (Aδ) and preganglionic sympathetic (B) fibres, the distribution of cold sensation blockade either mirrors or slightly exceeds that of the sympathetic blockade.^29^ Therefore, it is plausible that upper limb cholinergic sympathetic function was inhibited in 85% of the study participants.

The differential distribution of noradrenergic and cholinergic sympathetic blockade is a novel finding. Sudomotor skin responses have not previously been recorded concurrently with vasomotor skin responses during neuraxial blockade. However, a potential explanation for this unexpected result is preganglionic sympathetic fibre heterogeneity. Epidural analgesia affects efferent sympathetic function by interrupting axonal conduction in preganglionic sympathetic fibres as they traverse the epidural space.^30^ Historically preganglionic sympathetic fibres were classified homogenously as group B fibres (myelinated, conduction velocity 5–15 m s^−1^).^31^ More recently, however, at least four subgroups have been identified with differing conduction velocities, degrees of myelination, and neuropeptide contents.^32^ Group 1 and 2 fibres regulate cutaneous vasomotor function, whereas fibres containing corticotrophin-releasing factor control sudomotor function (group as yet undefined).^32^ At equivalent concentrations, local anaesthetics have differential effects on axonal conduction, dependent on fibre diameter, degree of myelination, and ion channel expression.^33^ Therefore, a potential explanation for the greater distribution of sudomotor sympathetic blockade is increased sensitivity to local anaesthetics in the cholinergic pathway preganglionic fibre subgroup.

As this was a real-world study of labour, body temperature was not standardised between visits, which in theory could have affected sympathetic skin response amplitudes. Core temperature was greater on the epidural visit, reflecting the heat stress of labour and the impact of labour epidural analgesia upon body temperature.^1^^,^^34^ There was no statistical difference in finger or toe skin temperature between visits. The impact of core temperature on sympathetic skin responses is not known, but local skin temperature is positively correlated with sudomotor skin response amplitude^35^ and negatively correlated with vasomotor skin response reduction rate.^17^ However, the lack of correlation between body temperatures and sympathetic skin response amplitudes in the present study suggests that the between-visit variation in body temperature did not have an impact on either the sudomotor or the vasomotor skin responses.

The results provide insight into a possible mechanism underlying ERMH: cutaneous sympathetic blockade. During heat stress, sudomotor skin response amplitudes are linked to evaporative (sweating) and non-evaporative (active vasodilation) heat loss.^7^^,^^19^^,^^36^ Labour is a form of heat stress in which contraction of uterine and skeletal muscle increases heat production up to 57%.^6^ As core temperature varies little during uncomplicated labour,^34^ heat loss must increase by a reciprocal amount, likely requiring active vasodilation and sweating.^7^^,^^8^^,^^11^ Because the trunk cutaneous sympathetic supply exits the vertebral column between the upper and lower limb supplies (T1–L2),^15^ this study suggests that labour epidural analgesia can inhibit regulation of cutaneous heat loss in up to 90% of the body surface area. Dysregulation of heat transfer on this scale increases the risk of heat imbalance, and during labour this could predispose women to an increase in body temperature.^37^ The impact of labour epidural analgesia on cutaneous heat loss has not been recorded directly. However, extension of labour epidural analgesia for intrapartum Caesarean section reduces cutaneous heat loss by 15%.^6^ Further research is required to explore the relationship between cutaneous sympathetic blockade and heat loss during labour epidural analgesia.

The heterogeneity in distribution of cholinergic sympathetic blockade between those who developed ERMH and those who did not is hypothesis generating. This study does not have a sufficient sample size to perform a formal subgroup analysis. However, all three participants who developed a temperature >38°C during labour had reduced sudomotor skin response amplitudes (>50%) in both the hand and foot. This was the case in only 71% of participants who had a normal core temperature throughout labour. Intermittent bolus labour epidural analgesia regimens are associated with a lower incidence of ERMH than continuous infusions, which may be owing to a smaller distribution of sympathetic blockade.^38^ The proportion of participants with reduced sudomotor skin response amplitudes (>50%) who did not develop elevated intrapartum temperature suggests that other factors also contribute to ERMH (e.g. baseline temperature and epidural duration).^37^ Therefore, repetition of this study with a larger sample is indicated to formally determine if cholinergic sympathetic blockade distribution is a risk factor for ERMH.

Strengths of this study are in its experimental design and ecological validity. Auditory evoked skin responses are the gold standard method of quantifying cutaneous sympathetic function during labour. Evoked skin responses are a superior method of assessing preganglionic sympathetic pathway integrity than simple recordings of skin blood flow, or sweat rate.^16^^,^^17^^,^^39^ Other methods of generating and recording evoked skin responses are painful (electrical stimulation, cervical magnetic stimulation) or invasive (microneurography) and, thus, are unsuitable for use in the perinatal period.^18^^,^^36^ Both sudomotor and vasomotor skin responses exhibit considerable inter-individual variation, but this source of error is mitigated by the crossover study design.^16^^,^^17^ Deployment of laboratory neurophysiological techniques in clinical setting enhances the ecological validity of the findings. However, result generalisability to other forms of labour epidural analgesia, such as patient-controlled epidural analgesia, cannot be guaranteed. A limitation is that it was not possible to eliminate emotional artifact from recordings. Sympathetic skin response central processing mechanisms are heavily influenced by the cerebral structures responsible for cognition and emotion.^17^^,^^40^ However, as subjectively the investigators observed that both the delivery suite and the postnatal ward generated similar quantities of emotional artifact, it is unlikely that this limitation led to a between-visit bias.

In summary, this physiological study investigated the impact of labour epidural analgesia on upper and lower limb cutaneous sympathetic function. Sudomotor function was inhibited in both the hand and the foot, whereas vasomotor function was inhibited in the great toe but not the index finger. During heat stress, the cholinergic sympathetic supply regulates cutaneous heat loss and, thus, cholinergic pathway blockade may increase the risk of hyperthermia during labour. Future research should examine the relationship between cholinergic sympathetic blockade distribution, cutaneous heat loss, and the risk of epidural-related maternal hyperthermia.

## Funding

Imperial Health Charity (RFPR2122_17; HFMM, CJM) and the Obstetric Anaesthetists’ Association (PHS, DAL, CJM).

## Authors' contributions

Study design: PHS, DAL, CJM

Data collection: GMVI, ARR, HFMM, PHS, CJM

Data processing and statistical analysis: GMVI, ARR, HFMM, CJM

Manuscript preparation: GMVI, CJM

Manuscript revision: all authors

## Declaration of interest

The authors have no conflicts of interest to declare.

## Appendix A Supplementary data

Multimedia components 1 Multimedia components 1
