# Supplementary material for: The cutaneous sympathetic blockade associated with labour epidural analgesia: a quasi-experimental study conducted during labour and after delivery
Source: Br J Anaesth. 2025 Sep 9;135(5):1231–9. doi: 10.1016/j.bja.2025.07.077 (PMC12597345; doi:10.1016/j.bja.2025.07.077)
Supplement: Multimedia components 1 [file mmc1.docx]

The cutaneous sympathetic blockade associated with labour epidural analgesia: a quasi-experimental study conducted during labour and after delivery.

**Authors:** Giulia M.V. Iacona; Aimee R. Rolph; Hugo F.M. Manteigas; Paul H. Strutton; David A. Low; Christopher J. Mullington^,^

**Supplementary material**

Contents

[Post-hoc power calculation: 2](#_Toc206091248)

[Supplementary Table 1: The differential distribution of cholinergic and noradrenergic sympathetic blockade. 2](#_Toc206091249)

[Supplementary Table 2: The relationship between body temperature, and sudomotor and vasomotor skin response magnitude. 2](#_Toc206091250)

# Post-hoc power calculation:

F tests - ANOVA: Repeated measures, within factors

Analysis: Post hoc: Compute achieved power

Input: Effect size f = 0.3725587

α error probability = 0.05

Total sample size = 20

Number of groups = 2

Number of measurements = 2

Corr among rep measures = 0.505

Nonsphericity correction ε = 1

Output: Noncentrality parameter λ = 11.2161604

Critical F = 4.4138734

Numerator df = 1

Denominator df = 18

Power (1-β err prob) = 0.8860556

# Supplementary Table 1: The differential distribution of cholinergic and noradrenergic sympathetic blockade.

|  | **Sudomotor > vasomotor** | **Sudomotor = vasomotor** | **Sudomotor < vasomotor** |
| --- | --- | --- | --- |
| Upper limb | 12 (60%) | 4 (20%) | 4 (20%) |
| Lower limb | 3 (15%) | 15 (75%) | 2 (10%) |

Values are n (%). SSR = sudomotor skin response; VSR = vasomotor skin response. Sudomotor > vasomotor: SSR amplitude epidural < control, but VSR amplitude epidural > control. Sudomotor = vasomotor: SSR amplitude epidural > control and VSR amplitude epidural > control, or SSR amplitude epidural < control and VSR amplitude epidural < control. Sudomotor < vasomotor: SSR amplitude epidural > control, but VSR amplitude epidural < control.

# Supplementary Table 2: The relationship between body temperature, and sudomotor and vasomotor skin response magnitude.

Correlations are shown for between-visit changes.

| ***Body temperature*** | ***Sympathetic skin response*** | ***Spearman rank coefficient (r_S_)*** | ***p-value*** |
| --- | --- | --- | --- |
| Core temperature | UL SSR amplitude | 0.37 | 0.14 |
|  | UL VSR reduction rate | -0.20 | 0.42 |
|  | LL SSR amplitude | -0.06 | 0.83 |
|  | LL VSR reduction rate | 0.30 | 0.24 |
| Finger temperature | UL SSR amplitude | -0.12 | 0.62 |
|  | UL VSR reduction rate | 0.11 | 0.64 |
| Toe temperature | LL SSR amplitude | 0.03 | 0.90 |
|  | LL VSR reduction rate | 0.17 | 0.50 |

UL = upper limb; LL = lower limb; SSR = sudomotor skin response; VSR = vasomotor skin response.
